# Clinical Research in Neonates: Redesigning the Informed Consent Process in the Digital Era

**DOI:** 10.3389/fped.2021.724431

**Published:** 2021-09-01

**Authors:** Evelien De Sutter, Birte Coopmans, Femke Vanendert, Marc Dooms, Karel Allegaert, Pascal Borry, Isabelle Huys

**Affiliations:** ^1^Clinical Pharmacology and Pharmacotherapy, Department of Pharmaceutical and Pharmacological Sciences, KU Leuven, Leuven, Belgium; ^2^Department of Development and Regeneration, KU Leuven, Leuven, Belgium; ^3^Department of Clinical Pharmacy, Erasmus MC, Rotterdam, Netherlands; ^4^Centre for Biomedical Ethics and Law, Department of Public Health and Primary Care, KU Leuven, Leuven, Belgium

**Keywords:** newborn, infant, health care professional, qualitative research, clinical trial, ethics, digital technology

## Abstract

**Background:** Currently, many initiatives are devoted to optimizing informed consent for participation in clinical research. Due to the digital transformation in health care, a shift toward electronic informed consent (eIC) has been fostered. However, empirical evidence on how to implement eIC in clinical research involving neonates is lacking.

**Methods:** Semi-structured interviews were conducted with 31 health care professionals active in Belgium or the Netherlands. All health care professionals had experience in conducting clinical research involving neonates. Interviews were audio-recorded, transcribed and analyzed using the framework method.

**Results:** Interviewees generally supported the use of eIC in clinical research involving neonates. For example, eIC could enable parents to receive study feedback via the eIC system. Requirements were expressed for parental involvement to decide on which feedback would be appropriate to return. Moreover, experts specialized in presenting information and designing electronic systems should be involved. Broad consensus among health care professionals indicates that the face-to-face-interaction between parents and the research team is vital to establish a relationship of trust. Therefore, it is necessary that the use of eIC runs alongside personal interactions with the parents. Concerns were raised about the accessibility of eIC to parents. For this reason, it was suggested that parents should always be given the possibility to read and sign a paper-based informed consent form or to use eIC.

**Conclusions:** Health care professionals' views indicate that the use of eIC in clinical research with neonates may offer various opportunities. Further development and implementation will require a multi-stakeholder approach.

## Introduction

Conducting clinical research in pediatric patients has been proven challenging due to economic, clinical and ethical considerations (1). As a result, these patients may receive medicines outside their marketing authorization, considering their limited clinical evidence pertaining to the efficacy and safety (2, 3). Over the past years, it has been widely acknowledged that medicinal development for pediatric populations is needed to protect their welfare (1). In 2007, the Pediatric Regulation came into force in the European Union, establishing a framework to support research and development of medicines for pediatric therapeutic needs (4). According to an ex-post evaluation, this Regulation has boosted the conduct of pediatric clinical research (5). In addition, the International Neonatal Consortium (INC) was established in 2015 under a grant from the US Food and Drug Administration (FDA) (6). The INC, a global collaboration of key stakeholders such as regulatory agencies, academic researchers, neonatal advocacy groups, pharmaceutical companies and neonatal nurses, aims to foster the therapeutic toolbox for neonates (7). Unique challenges are related to the conduct of neonatal research. For example, parental decision-making may be impaired when urgently enrolling a neonate with life-threatening conditions in a research study. Therefore, parents could be approached in the antenatal period to convey information on postnatal study enrolment (8). From an ethical point of view, each participant involved in clinical research needs to be adequately informed during the informed consent (IC) process about what the research study entails. This process is vital to safeguard the participants' well-being, health and rights (9). When research is conducted in neonates, the IC process needs to inform their legally designated representative(s), often the parents, enabling them to decide on participation in the best interest of their child (10). In the Netherlands, both parents need to sign the IC (11). Similarly, both parents need to sign the IC in the United States, except if the institutional review board considers the permission of only parent sufficient. This may concern clinical investigations that do not involve greater than minimal risk or minimal risk with the prospect of direct benefit for the child (12). However, conflicting approaches to Belgian legal instruments such as the Civil Code and the Law of 7 May 2004 on experiments on human beings emerged (13, 14). Based upon article 373 of the Civil Code, some suggest that only one parent has to provide IC if both parents are living together. According to the Law of 7 May 2004, IC needs to be obtained from both parents, on the condition that parents have joint custody of their child. As a golden mean, some advice to obtain the signature of at least one parent at the time of recruitment and the written authorization of the other parent along the conduct of the study (15). In the United Kingdom, the IC from only one parent is required (16).

However, obtaining IC from parents whose neonate is critically ill may be challenging (3, 17, 18). For example, parents can feel emotionally distressed or the mother can experience interfering medical conditions such as preeclampsia or post-anesthesia (3, 17, 19). Parents are often overwhelmed and may have a poor understanding of the study-related information when being under time pressure (18, 20). As a result, they may feel incapable to decide about their neonate's participation in a research study (20). Key points suggested to improve the IC process are antenatal awareness and discussion, training of those seeking IC, continuing two-way communication and clear and well-regulated information (20–22). Related to these key points, advances in technology have resulted in new opportunities to enhance the paper-based IC process (23). According to the FDA, electronic informed consent (eIC) is defined as “*The use of electronic systems and processes that may employ multiple electronic media, including text, graphics, audio, video, podcasts, passive and interactive Web sites, biological recognition devices, and card readers, to convey information related to the study and to obtain and document IC*” (24). eIC offers the possibility to present information in a more informative and engaging way by using multimedia tools (25). Moreover, an eIC system, allowing parents to manage their eIC, could enable researchers to remain in contact with parents over time. For example, study results can be provided to the parents on regular basis, parents can revisit their consent decision or repeated IC discussions can be set up. Additionally, an eIC system could be tailored to the parents' needs and to the research endeavor (26, 27). Nevertheless, stakeholders, such as research participants and researchers, voiced concerns about access of eIC to specific population groups and data privacy (25).

Currently, paper-based IC is still widely used in neonatal research. Less attention has been given to the implementation or use of eIC in clinical research involving neonates (28, 29). For this reason, this study aims to investigate health care professionals' (HCPs) perspectives, as one of the relevant group of stakeholders, on the current paper-based IC process and how this process could be improved by using eIC. Moreover, this study investigates how an eIC system should be designed to enable responsible implementation in clinical research in neonates. The following research questions are addressed in this study:

- What are HCPs' views on the current IC process?
- What are potential advantages and disadvantages of using an eIC system in clinical research involving neonates?

## Methods

### Participant Selection and Recruitment

Semi-structured interviews were conducted with HCPs (i.e., physicians and nurses) from both university and non-university hospitals located in Belgium and the Netherlands. Participants were selected through a combination of purposive sampling and snowballing. Purposive sampling implies the identification and selection of potential participants who are able to provide insights into the topic of interest (30). Additionally, snowballing sampling was used whereby potential recruits were identified from suggestions made by existing interviewees. Participants fulfilled the inclusion criteria when they were experienced in seeking parental (electronic) IC for a neonate's participation in clinical research, were fluent in English or Dutch and were active in Belgium or the Netherlands. An invitation mail was sent to suitable participants, including the IC of the interview study and the interview guide (Supplementary Material I). All participants provided IC prior to participation in this study. Participants were recruited until data saturation was reached.

### Conduct

An interview guide was derived based on the research questions and a systematic literature review (25). At the time of conducting this review, no empirical literature was available with regard to the use of eIC in neonatal research. Therefore, it needs to be investigated how an eIC system can be effectively adapted to parents' and HCPs' needs and how ongoing communication with parents can be established during and after a research study (25). The interview guide was piloted in two interviews and consisted of questions related to the IC process and specifically, the use of paper-based IC forms and eIC. The pilot interviews, conducted with HCPs, were not included in the qualitative analysis. Moreover, open discussion was encouraged to gain an in-depth understanding of interviewees' perspectives. All interviewees received the interview guide in advance in order to prepare themselves for the interview. Interviews were conducted via teleconference from October 2020 until January 2021. Interviews were 30–60 min long. At the start of each semi-structured interview, an electronic slideshow was used to introduce the interviewer, the aim of the study and to provide the definition of eIC, as issued by the FDA (24). This definition was used to inform interviewees that both conveying information and obtaining parents' signature occur via electronic means when using eIC. Interviews with Belgian and Dutch HCPs were conducted by two researchers (BC and FV, respectively) and were supervised by another researcher (EDS). All interviews were conducted in Dutch and were audio-recorded. Moreover, Dutch quotes were translated to English upon inclusion in the manuscript.

### Analysis

The qualitative data analysis utilized a thematic analysis framework (31). Researchers (BC and FV) immersed themselves in the data by transcribing the interviews, reading the transcripts and re-listening to the audio-recordings. The first two transcripts were coded independently by three researchers (BC, EDS and FV) and were subsequently compared. Interviews were coded deductively based upon themes of the interview guide. Additionally, inductive codes were created when critically observing the data. Deductive and inductive codes were grouped into broader categories in order to create a coding tree using NVivo software (Supplementary Material II). This coding tree was then applied independently to the other transcripts by two researchers (EDS and FV or BC). The coded data were compared and were charted in a framework matrix. Hereafter, the data obtained were interpreted, outcomes were described and quotes of individual interviewees were added for further clarification.

## Results

In total, 31 interviews were conducted. An approximately equal number of HCPs active in Belgium and the Netherlands took part in this qualitative study (Table 1). Results of the interviews are structured according to the categories identified during analysis: involvement of parents during the IC process, current paper-based IC process and eIC process.

**Table 1:** Descriptive characteristics of the interviewees.

**Characteristics**	**Number of interviewees**
**Country**	
Belgium	16
The Netherlands	15
**Stakeholder group**	
Neonatologist	25
Pediatrician	3
Study nurse	3

### Involvement of Parents During the IC Process

Generally, HCPs asserted that parents are involved during the IC process by discussing the study information with the HCP.

“*It is marvelous how parents, whose neonate is fragile, are willing to participate in a research study.”* (HCP-BE 4)

Nevertheless, according to the majority of HCPs, some parents make the decision for their neonate to partake in a study based on their relationship of trust with the HCP rather than deliberative weighing of the study information. It was indicated that parents' involvement in the IC process is negatively influenced by a low socioeconomic status and an inadequate understanding of study information. Moreover, the majority of HCPs believed that the timing of seeking IC is not optimal. Distressed and vulnerable parents, who are already overloaded with questions and fear regarding their child's health, may not always have the capacity to process study information. Therefore, HCPs aim to focus on antenatal counseling to facilitate an informed decision, in particular when women have a high risk of premature birth. Even then, seeking IC can be overwhelming to parents.

“*Parents are often shocked by the premature birth of their child, next to a medical problem for which the child has to be treated*.” (HCP-BE 12)

From an ethical perspective, Belgian HCPs consider it of utmost importance that both parents sign the IC form. It was raised that collecting parents' signatures is the best way to ensure dual parental permission for their neonate to participate in clinical research. However, almost all HCPs argued that gathering both signatures is often practically challenging. For example, when fathers need to resume work after their paternity leave.

“*If it concerns a research study somewhat later in the disease process, we experience that fathers already go back to work and therefore, it is challenging to obtain their signature.”* (HCP-NL 2)

It was considered essential that HCPs convey information pertaining to a research study to both parents, in particular if it concerns an interventional study. An interventional study is associated with various uncertainties with regard to the benefits and the long-term risks. Therefore, it is of great importance that both parents are adequately informed by the HCP to enable a well-informed decision on their neonate's study participation. One HCP believed that if parents would sign the IC separately, they would be less influenced by each other's decision. Nevertheless, HCPs took it for granted that both parents act in their child's best interest.

### Current Paper-Based Informed Consent Process

Overall, HCPs considered the IC process as a valuable approach of informing parents about a research study. The majority of interviewees recognized that the face-to-face contact between parents and the responsible HCP is vital to establish a relationship that involves trust. Some HCPs voiced that the decision to partake in a research study is based upon the oral explanation of study information provided by the HCP rather than by thoroughly reading the paper-based IC form.

“*When orally explaining the study to parents, I am able to personalize the information based on parents' needs.”* (HCP-NL 4)

Nevertheless, concerns were voiced that parents may be subjected to undue pressure to freely decide on their neonate's study participation. Because the responsible HCPs are often the researchers, their roles are conflated. Additionally, HCPs are able to determine from nonverbal language whether parents are adequately informed and are able to provide truly IC. If parents would have additional questions, HCPs can immediately answer these during the face-to-face contact.

The majority of HCPs agreed that the written, paper-based IC form contains all study details. For this reason, it enables parents to review study information on their own pace. Nevertheless, it was argued that IC forms are very long and scientific and legal in nature and, therefore, often incomprehensible.

“*I notice that some IC forms are very extensive. I experience that parents often do not read the full document*.” (HCP-BE 3)

Another challenge raised by HCPs is that a paper-based IC form often gives rise to improper storage and documentation, such as signing incorrect IC versions. Moreover, IC forms need to be manually scanned into the neonates' electronic health record. In the end, parents often need to be recontacted to provide or to ask for further consent, due to new research requirements, and this is considered an administrative burden on the research team. One HCP raised the point that when parents are recontacted after finishing the research study, some do not remember that their child took part in the study.

### Electronic Informed Consent Process

Only 1 HCP has obtained practical experience with eIC in view of an observational study whereas 3 other HCPs have gathered experience with using multimedia to inform parents about a research study.

#### Potential Advantages and Disadvantages

HCPs agreed that an eIC system could inform parents in an interactive way. For example, a question and answer session could be implemented to assess parents' level of understanding. Moreover, the use of multimedia was considered a distinct benefit to convey information. By implementing video or graphics, information can be made visually attractive. HPCs believed that an eIC system could support data integrity by guaranteeing that eIC is correctly managed. For example, an audit trail could be integrated to log who performed a certain action and when. In addition, it was believed that data stored in the eIC system could be automatically exchanged with the electronic health record, and thus, adequately stored.

Some HCPs thought that the use of eIC would result in less personal interaction with parents. Therefore, it was raised that the eIC process must be a combination of personal contact and electronically reviewing study information and signing the eIC. Some HCPs highlighted that remotely signing eIC could facilitate obtaining both parents' signatures. Moreover, telemedicine technology could be integrated enabling to remotely video conference with parents.

“*Personal contact helps to explain the study details and which value the study may have for the neonate.”* (HCP-NL 5)

Moreover, parents need to have the opportunity to ask questions related to the research study. Several HCPs remarked that parents could indicate, via the eIC system, that they have further questions. Based on the type of questions, HCPs could choose to answer these questions remotely or during a physical consultation. One HCP indicated that it would not be feasible to quickly respond when parents would be worried or if information would be unclear.

Generally, HCPs did not experience a major impact of the COVID-19 pandemic on the IC process. Several HCPs mentioned that they inform the parents about their neonate, who receives inpatient neonatal care, on a daily basis. For this reason, there is no need to schedule additional contact moments with parents to discuss research study information.

“*Neonates who are involved in a research study are very often hospitalized in the neonatal intensive care unit so there is already face-to-face contact with the parents. As a result, COVID-19 did not have a negative impact on the IC process*.” (HCP-BE 9)

However, when the mother was infected with COVID-19, IC was obtained by phone or signed IC forms were stored in sealed plastic bags and processed several days after signing. In addition, only HCPs who took care of the neonate were allowed to visit the unit and, thus, other HCPs who conducted research were not able to have a face-to-face conversation with parents. Therefore, HCPs indicated that it would be beneficial to organize the IC process via an electronic device. Another HCP raised that eIC could facilitate providing information to parents, during and after the COVID-19 pandemic. It was believed that the pandemic has driven fundamental change in health care delivery.

“*Before the COVID-19 pandemic, the use of telehealth was not common in practice due to several barriers and now it turns out that it is widely adopted. It will be the same with eIC*.” (HCP-NL 13)

Concerns were raised about the accessibility of eIC to parents. One HCP mentioned that an unfavorable socioeconomic environment of the pregnant mother is an important determinant for premature delivery. As a result, some women who are admitted to the neonatal intensive care unit will lack digital literacy.

“*It is important that parents who do not have access to the Internet are also able to let their child participate in a research study*.” (HCP-BE 16)

Therefore, HCPs believed that alternative solutions need to be foreseen, such as a paper-based IC form or electronic devices which can be offered to parents to review study information. Moreover, concerns were reported about how eIC should be submitted to the ethics committees.

#### Accessibility to an Electronic Informed Consent System

HCPs were asked how long an eIC system should be available to parents. Their key concerns and needs with regard to the accessibility to an eIC system included the following:

- If the eIC system is used only to inform parents and to obtain their consent, it should be accessible until the final analysis of research data. If the system is also used to facilitate the establishment of a long-term interaction with parents (e.g., to provide results), it should be longer accessible.
- The accessibility depends on the type of research study. For example, if it concerns a study with a long follow-up period, the eIC system should be longer accessible in comparison with a study without follow-up.
- There should be a contact point in case parents would have questions after the end of the research study.
- If maintenance of the eIC system is too costly, parents should have the option to download data and store these data themselves.
- Children have the right to know in which study they have taken part. Therefore, they should have the possibility to retrieve information about the study and the study outcomes. For example, if a subject has medical issues several years after study participation, it can be valuable to access the eIC system to refresh knowledge about the study.

Additionally, some HCPs mentioned that it would be valuable to understand the frequency parents, or the study subjects themselves, access the eIC system after participation in the research study.

#### Interactive Informed Consent

##### Long-Term Interaction

As reported by HCPs, an eIC system could facilitate maintaining contact with parents during or after the research study. It was considered a great opportunity to provide feedback on the study, such as study results, via an electronic device. Establishing a longitudinal interaction between parents and the study team may benefit transparent communication. According to HCPs' experiences, parents are willing to receive the general study results as well as the individual results of their neonate. One HCP raised that disclosing results is still not a common practice, although parents have the right to receive these. It was also mentioned that individual results related to a neonate should be conveyed during a clinical consultation, in particular if it concerns sensitive information.

Many HCPs indicated that the principal investigator should, together with the ethics committee, decide which results can be shared. Moreover, it was considered important to involve parent representatives to discuss which information may be relevant and appropriate to share during or after the research study. HCPs were convinced that information must be returned in lay language. It was also indicated that the use of an eIC system must not be at the expense of face-to-face conversations with parents to further explain the results.

“*It is of utmost importance that parents think together with HCPs and members of the ethics committee about which information can be returned. Moreover, feedback can be asked to parents on how the eIC system can be improved*.” (HCP-NL 11)

Because an eIC system could facilitate continued contact with parents, they can be easily recontacted if additional consent needs to be obtained due to unforeseen circumstances. Moreover, the system could enable parents to complete surveys during the course of the study. Parents may receive a pop-up alert indicating that new information is available or that surveys need to be completed.

##### A Personalized Interface

HCPs were of the opinion that a personalized interface is meaningful for both the parents and the HCPs. Interviewees were asked how they would personalize an eIC interface. Opportunities for adapting the interface to parents', study subjects' and HCPs' needs were identified (Table 2).

**Table 2:** Overview of opportunities for a personalized eIC interface according to HCPs.

Parents could be able to: •Choose the language in which they would like to review study information •Choose the format in which they would like to review study information, e.g., visuals, graphics, text only •Access additional information if preferred. For example, hyperlinks to official websites may be implemented. •Receive a copy of eIC, e.g., by email •Set preferences on how they wish to communicate with HCPs. For example, parents may be able to indicate if they would like to discuss study information with the HCP in person or remotely. •Set preferences to establish a long-term interaction •Complete a question and answer session to assess their comprehension. Parents can be redirected to the information related to the topic that was incorrectly answered.
HCPs could be able to: •Check if parents read the study information
Study subjects could be able to: •Have an overview of clinical studies in which they have previously taken part

Few HCPs questioned whether information could be adapted to parents' education level. Nevertheless, concerns were raised about the ethical and practical applicability. Parents in the neonatal intensive care unit may experience psychological and emotional distress. Therefore, some HCPs advised to develop an eIC system including user-friendly information.

“*Not every woman who has given birth will be motivated to use eIC and to review complex information*.” (HCP-NL 7)

For this reason, some HCPs recommended to include diverse experts specialized in presenting information and designing interfaces.

It was widely agreed that all parents need to receive the same amount of information. Based upon their preferences, they can decide to access additional information. Parents could also be able to indicate preferences to establish a long-term interaction. For example, they could choose to receive study results, information about the study status or updates to eIC via an electronic device.

“*Some parents will not look back at the eIC system, while others are really interested. If they are informed about the progress of the study, it will improve study involvement*.” (HCP-BE 5)

### Implementation and Hosting

Several HCPs mentioned that an eIC system should be compatible with various systems already integrated in the hospital infrastructure. Because hospitals may use different technologies, integration was considered challenging. Moreover, it was raised that the practical implementation of an eIC system would be an important factor to have a successful and well-used system. Therefore, relevant stakeholders should be involved to learn about their practical considerations regarding such a system.

The majority of HCPs indicated that the study team should be responsible for hosting an eIC system. However, the end responsibility is with the principal investigator. Moreover, technical support should be offered when required. Some other HCPs believed that the hospital itself or an independent party must be responsible for hosting the system. It was also indicated that it would be better to have a European initiative.

“*It would be better to have a European approach. In this way, there is no need to use systems that work differently, especially when multicentric studies are conducted*.” (HCP-BE 8)

## Discussion

This qualitative study aimed to assess the opinions of Belgian and Dutch HCPs involved in clinical research in neonates with regard to the current paper-based IC process and a potential shift to eIC. Belgium has, across European countries, one of the highest number of clinical trials per inhabitant (32, 33). Also in the Netherlands many clinical trial applications are submitted to the relevant regulatory authorities yearly, especially in comparison with its neighboring country Germany (33). Generally, Belgian and Dutch HCPs were satisfied with the current paper-based IC process to inform participants about several aspects of a research study. Drawbacks were related to the complex terminology used in an IC form and documentation and storage challenges. Potential advantages of eIC, suggested by HCPs, included the possibility to use multimedia to convey study information, the facilitation of data integrity and the exchange of data with the electronic health record. Moreover, an interactive eIC system, enabling a personalized approach and establishing longitudinal interactions, was valued highly by the HCPs. However, some HCPs were concerned about the accessibility of eIC to parents and the impact of eIC on the personal interaction with parents.

### Implementation of Electronic Informed Consent

Only 1 interviewed HCP had gathered practical experience with eIC. The question may raise which challenges hinder the widespread adoption of eIC in clinical research involving neonates. Next to these reported by HCPs, such as the use of eIC by parents who lack digital literacy, other challenges could be in place. For example, compliance with national requirements could be challenging. In the Netherlands, the Medical Research Involving Human Subjects Act states that a written, dated and signed IC is needed to participate in scientific research (34). This Act is currently under revision to include the use of electronic signatures, which is currently not yet allowed (35). On a Belgian level, a guidance related to the use of eIC in interventional clinical trials was recently issued (36). The creation of this guidance was coordinated by the Clinical Trial College, an independent body within the Federal Public Service Health, Food Chain Safety and Environment (37), and developed in collaboration with representatives of the Belgian Association of Research Ethics Committees, the Belgian Association of the Innovative (Bio)pharmaceutical Industry and patient organizations. This guidance provides a framework when using eIC in interventional clinical trials. For example, the research subject should always have the choice between signing a paper-based IC form or signing electronically (36). Another challenge could be related to the compatibility of eIC systems to interface with software already implemented in the research sites. Resources must be invested to strive for interoperability, which may be costly for a site. Interoperability allows two or more systems to electronically exchange data, and is considered essential for digital innovations for future medicine (38).

### Parental Involvement

Interviewees widely agreed that parents prefer to receive general results related to the research study and individual results relevant to their neonate, which is in line with the available literature. Greenberg et al. found that parents appreciate the return of results of the study in which their child has taken part (39). According to interviewed HCPs, parents can be involved to decide on relevant and appropriate information to be shared via an eIC system. The European Foundation for the Care of Newborn Infants (EFCNI) recommends to involve parent representatives during the research process to learn from their experiences related to a specific disease or service. The EFCNI strives to collaborate with parent representatives in various, consecutive phases of the research process, from study protocol development until the dissemination of research results (40). In addition, the INC aims to inform clinical research by collecting the perspectives of parents who have experience with participation of their neonate in a research study (41). Some HCPs were of the opinion that user-friendly information should be used to inform parents. Therefore, it is essential to involve parents, next to experts in human-computer interaction, when setting up an eIC system. For example, it is important that the information will not overwhelm already traumatized parents whose neonate is fragile. In addition, HCPs believed that the research site needs to foresee electronic devices which parents can use to review study information and electronically sign the IC. The use of digital technologies could result in the creation of digital vulnerable groups, also called the digital divide (42). Therefore, it is crucial that the usability and the user-friendliness are adequately assessed and improved by involving parents in the design of eIC, considering their education level or health literacy (43).

### A Dynamic System

HCPs mentioned that antenatal counseling is of great value to support an informed decision. Golec et al. confirm that strengthening continuous communication with parents is important. It would be optimal to start the IC process early on and communicate until after the intervention (8). Another study, researching parents' views of whom their child has taken part in longitudinal studies, found that parents would like to regularly receive information during the conduct of the study (44). HCPs expect that an eIC system could facilitate ongoing communication with parents during or after the research study. An eIC system, enabling dynamic interactions over time, can update parents about the outcomes of the study or can invite them for a clinical consultation (27). In addition, it is considered important that parents are continuously educated about the study details and their rights (8). When long-term contact is established between parents and the research team via an eIC system, it could help to remind parents of their child's participation in a research study, which may be forgotten (45). As formulated by the interviewed HCPs, it could be up to the parents to indicate how they would like to receive information. The eIC interface should give parents sufficient control to adapt it based on their needs, while being cautious to avoid burdensome practices (46). HCPs indicated that the trusting relationship with parents is one of the main reasons to consent, which is in line with the available literature. For example, it was reported that information written in the IC form did not always support parents' understanding (47). Similarly, HCPs raised that IC documents are often difficult to understand, due to scientific and legal information. HCPs have a legal and ethical duty to convey the necessary information to parents (9, 10). By using eIC, the same information needs to be provided to parents. However, this information can be provided by using various multimedia formats, as indicated by the interviewed HCPs. This finding was also observed in a systematic review. According to some research participants, multimedia could be more effective to convey research information in comparison to written text. Additionally, the use of simple, concise language and the implementation of a question and answer session were recommended to support parents' understanding (25).

### The Use of Electronic Informed Consent During the COVID-19 Pandemic

According to HCPs, eIC could be beneficial during the COVID-19 pandemic. For example, eIC decreases the risk of being exposed to the virus and the use of personal protective equipment that needs to be used when approaching COVID-19 infected parents in the course of the IC process (48). The Central Committee on Research Involving Human Subjects, a competent authority in the Netherlands, issued a guidance on conducting clinical research during restrictive measures due to the COVID-19 pandemic (49, 50). This guidance states that, if study medication is directly sent to research subjects' home, the subjects need to provide IC for the use of their personal information. IC can be provided orally and confirmed by mail (49). The Belgian guidance, published by the Federal Agency for Medicines and Health Products, mentions that “*Any validated and secure electronic system already used in the investigation for obtaining informed consent can be used as per usual practice and if in compliance with national legislation*” which is in line with the guidance issued by the European Medicines Agency (51, 52). Moreover, other countries issued guidance about the management of clinical trials during the COVID-19 pandemic. For example, Hungary explicitly states that the use of eIC is forbidden (53). The FDA guidance, on the Conduct of Clinical Trials of Medical Products During the COVID-19 Public Health Emergency, refers to eIC as a valuable approach to obtain the IC of a hospitalized patient who is in isolation (54).

### Strengths and Limitations

To our knowledge, this study is the first seeking to investigate the perspectives of Belgian and Dutch HCPs with regard to eIC in clinical research involving neonates. A strength of our study is that interviewed HCPs were equally recruited from Belgium and the Netherlands. Interviews were conducted by two junior researchers (BC and FVE) but were supervised by another researcher (EDS) who had previous experience in conducting semi-structured interviews. To minimize variability between interviews, an interview guide was used. The design of this interview guide was informed by the conduct of a systematic literature review. Additionally, the trustworthiness of the interview study was enhanced by two researchers who independently coded all interviews.

Qualitative research does not aim to provide results with a universal validity, but rather to provide insights into HCPs' perspectives about (electronic) IC. Therefore, caution should be taken when generalizing results. Selection bias might have occurred because the majority of HCPs were contacted by using the research group's network. Moreover, it may be possible that HCPs who are more interested in eIC decided to participate in this interview study. Only 1 interviewed HCP did have experience with eIC. Other perspectives may arise if HCPs would use eIC in practice. However, we were able to gain insights in how stakeholders expect that eIC would influence the conduct of clinical research involving neonates.

### Future Perspectives

This qualitative study provides insights into HCPs' perspectives on the use of eIC in clinical research involving neonates. Future research should focus on investigating parents' views to better understand the value of eIC in a stressful situation. Furthermore, attention must be paid to how study information can be effectively communicated to parents. Participatory design can be used to involve parents throughout the design process of an eIC system to better understand their needs. By involving parents in the design, the system will be co-created and the potential for success will be more likely.

## Conclusion

Overall, Belgian and Dutch HCPs were positive toward the implementation of eIC in clinical research involving neonates. Ideally, the eIC process should be a combination of face-to-face conversations with a HCP and electronically reviewing research information and providing consent. Moreover, opportunities for a personalized interface were identified, as well as HCPs' views and concerns regarding a longitudinal relationship with parents during and after a research study. However, the implementation of eIC requires further research and goes hand in hand with the involvement of parents themselves.

## Data Availability Statement

The datasets presented in this article are not readily available because participants did not provide consent to share their transcripts with parties other than the researchers. Requests to access the datasets should be directed to Evelien De Sutter, Evelien.desutter@kuleuven.be.

## Ethics Statement

The studies involving human participants were reviewed and approved by the Ethics Committee Research UZ/KU Leuven. The participants provided their written informed consent to participate in this study.

## Author Contributions

EDS, FV, and BC designed the interview guides. The interview guides were reviewed by MD, KA, and IH. Interviews were conducted by FV and BC and supervised by EDS. Data analysis was conducted by FV, BC, and EDS. EDS wrote the first draft, which was revised and reviewed by FV, BC, MD, KA, PB, and IH. All authors approved the final manuscript.

## Conflict of Interest

The authors declare that the research was conducted in the absence of any commercial or financial relationships that could be construed as a potential conflict of interest.

## Publisher's Note

All claims expressed in this article are solely those of the authors and do not necessarily represent those of their affiliated organizations, or those of the publisher, the editors and the reviewers. Any product that may be evaluated in this article, or claim that may be made by its manufacturer, is not guaranteed or endorsed by the publisher.
