# Supplementary material for: Clinical Research in Neonates: Redesigning the Informed Consent Process in the Digital Era
Source: Front Pediatr. 2021 Sep 1;9:724431. doi: 10.3389/fped.2021.724431 (PMC8441012; doi:10.3389/fped.2021.724431)
Supplement: Supplementary file 1 [file Data_Sheet_1.pdf]

SUPPLEMENTARY MATERIAL I – INTERVIEW GUIDE

I. Introductory questions

1. To what extent do you have experience with clinical research involving neonates?
2. What is your experience with parents' involvement in the informed consent process?
3. What was/is the impact of the COVID-19 pandemic on the informed consent process?

II. Questions regarding the paper-based informed consent process

1. What is your experience with the paper-based informed consent process?
   a) What are the positive aspects?
   b) What are the negative aspects?

III. Questions regarding the electronic informed consent process

1. To what extent do you have experience with electronic informed consent?

The following questions (2-5) apply only to health care professionals with practical electronic informed consent experience:

2. How does the use of electronic informed consent influence the clinical research process in comparison to a paper-based consent process?
3. How is the electronic informed consent system designed?
   a) Do you have any recommendations? Which one(s)?
   b) Do the parents receive personalized information?
   c) What is your opinion regarding personalization?
   d) What are the advantages and disadvantages of personalization?
   e) If relevant, how would you personalize the system?
   f) Who manages the system?
4. What is your opinion regarding the return of relevant information of a clinical research study to parents using the electronic informed consent system?
   a) Which information may be shared with the parents via the electronic informed consent system?

b) Who do you think should decide what kind of information may be shared via the electronic informed consent system?

5. Did you experience any barriers in the implementation of electronic informed consent in this clinical research study/studies? Which one(s)?

<u>The following questions (2-7) apply only to health care professionals who do not have experience with electronic informed consent:</u>

2. What could be potential benefits or drawbacks when using an electronic informed consent system?
3. About the design of an electronic informed consent system:

   a) Which elements are needed to create an interactive electronic informed consent system?

   b) What is your opinion regarding personalization of the system?

   c) What are the advantages and disadvantages of personalization?

   d) If relevant, how would you personalize the system?
4. What is your opinion regarding the return of relevant information of a clinical research study to parents using the electronic informed consent system?
   a) Which information may be shared with the parents via the electronic informed consent system?
   b) Who do you think should decide what kind of information may be shared via the electronic informed consent system?
5. Which barriers or challenges may be experienced in the implementation of electronic informed consent in clinical research?
6. Who do you think is best placed to manage the system?
7. How long should the system be available to parents?

To end:

- Do you have any further comments or issues which you think may be relevant to the design, implementation, or use of an electronic informed consent system?
- Do you have a suggestion for other interviewees?
- Do you have any questions?

SUPPLEMENTARY MATERIAL II – CODING TREE

Code	Sub-code level 1	Sub-code level 2	Description
Involvement of parents during the IC process	Obtaining parents' signatures		What is the opinion of the interviewee on the involvement of parents during the IC process?
	Conveying information to parents		
	Informed decision		
Current paper-based IC process	Challenges		What are the disadvantages of the paper-based IC process?
		Cognitive burden	
		Long-term interaction	
		Documentation and storage	
	Advantages		What are the advantages of the paper-based IC process?
		Detailed information	
		Face-to-face contact	
eIC process	Experience		Does the interviewee have experience with eIC?
	Advantages		What are the (potential) advantages of the eIC process?
	Challenges		What are the (potential) challenges of the eIC process?
	Managing the platform		Who is best placed to manage the eIC platform?
	Personalization		- What is the opinion of the interviewee on personalization?<br>- How can an eIC system be personalized?
	Long-term interaction		How can eIC establish long-term interactions?
		Recontacting	
		Communication of study results	
	Accessibility		How long should the eIC system stay available to parents?
	Design of the platform		Which elements related to the design of eIC are important?
	COVID-19		- What is the impact of the COVID-19 pandemic on the IC process?<br>- What is the potential of eIC during the COVID-19 pandemic?
